# IntAssoPlot: An R Package for Integrated Visualization of Genome-Wide Association Study Results With Gene Structure and Linkage Disequilibrium Matrix

**DOI:** 10.3389/fgene.2020.00260

**Published:** 2020-03-20

**Authors:** Fengyu He, Shuangcheng Ding, Hongwei Wang, Feng Qin

**Affiliations:** ^1^Engineering Research Center of Ecology and Agricultural Use of Wetland, Ministry of Education, Agricultural College, Yangtze University, Jingzhou, China; ^2^Hubei Key Laboratory of Waterlogging Disaster and Agricultural Use of Wetland, Agricultural College, Yangtze University, Jingzhou, China; ^3^Hubei Collaborative Innovation Center for Grain Industry, Agricultural College, Yangtze University, Jingzhou, China; ^4^College of Biological Sciences, China Agricultural University, Beijing, China

**Keywords:** genome-wide association study, gene structure, LD, visualization, linking line, integration

## Abstract

Genome-wide association study (GWAS), exploring the historical and evolutionary recombinations at the population level, is a major method adopted to identify quantitative trait loci (QTL) for complex traits. However, to summarize GWAS results, gene structure, and linkage disequilibrium (LD) in a single view, multiple tools are required. It is tedious to generate these three results and manually put them together; moreover, it may eventually lead to inaccuracies. On the other hand, genotype markers are usually detected by DNA- and/or RNA-Seq. For GWAS analysis based on RNA-Seq, markers from DNA-Seq provide more genetic information when displaying LD. The currently released software package does not have this function for an integrated analysis of LD, using genotypic markers different from that in association analysis. Here, we present an R package, IntAssoPlot, which provides an integrated visual display of GWAS results, along with LD and gene structure information, in a publication-ready format. The main panel of an IntAssoPlot plot has a connecting line linking the genome-wide association *P*-values on the -log_10_ scale with the gene structure and LD matrix. Importantly, IntAssoPlot is designed to plot GWAS results with LD calculated from genotypes different from those in GWAS analysis. IntAssoPlot provides a powerful visualization tool to gain an integrated insight into GWAS results. The functions provided by IntAssoPlot increase the efficiency by revealing GWAS results in a publication-ready format. Inspection of the output image can provide important biological information, including the loci that passed the genome-wide significance threshold, genes located at or near the significant loci, and the extent of LD within the selected region.

## Introduction

During the past few years, a sufficient number of molecular markers and availability of fast and accurate variance component estimation methods have made GWAS an ideal tool to detect genetic relationships with complex traits (Mackay, 2001; Yu and Buckler, 2006; Wang and Qin, 2017). However, it is difficult to summarize GWAS results, genomic context, and LD in a single and publication-ready view. Generally, researchers use Haploview (Barrett et al., 2005) and plink (Purcell et al., 2007) for LD analysis and visualization, CSDS (Hu et al., 2015) and Phytozome (Goodstein et al., 2012) for gene structure visualization, and TASSEL (Bradbury et al., 2007) and GAPIT (Lipka et al., 2012) for visualizing associations. Subsequently, the images generated are compiled together by manual copy and paste steps.

To efficiently visualize GWAS results, packages such as LocusZoom, cgmisc, Ldlink, and Assocplots have been developed (Pruim et al., 2010; Kierczak et al., 2015; Machiela and Chanock, 2015; Khramtsova and Stranger, 2017). LocusZoom is a web-, Java- and R-based tool, Ldlink is a web-based tool, and cgmisc and Assocplots are stand-alone tools based on (Ihaka and Gentleman, 1996) and Python (Sanner, 1999), respectively. Ideally, the tools for visualizing GWAS results should represent information detailing (1) the loci passing the genome-wide significance threshold, (2) the genes present at or near the significant loci, and (3) the linkage disequilibrium (LD) structure of the significant loci. In order to display the information listed above, the function plot.manhattan.genes, in the package cgmisc, arranges the relative plots together in a single page. Even though the LocusZoom and cgmisc can display regional GWAS information, such as the association of signal relative to genomic position and LD (LD between the most significant associated loci with the rest), no connecting line linking the significant loci, gene structure, and LD matrix is shown. Furthermore, single nucleotide polymorphism (SNP) genotypes detected by RNA-Seq are usually located in the coding and transcriptional regulatory regions [16], which cannot fully represent the genomic variation. Therefore, for the GWAS results calculated from SNPs detected by RNA-Seq, it is necessary, though difficult, to display GWAS results with LD calculated from genome-wide SNPs by re-sequencing.

We, therefore, developed an R package called IntAssoPlot, which would not only display the relative significance loci, gene structure, and linkage disequilibrium matrix derived from marker dataset (which is same or different from that for GWAS), but would also draw a connecting line to link them in one panel without the need for further manual arrangement. Additionally, IntAssoPlot is designed to show GWAS results at a single gene level, displaying not only information related to significant loci and linkage disequilibrium structure, but also that related to the detailed genetic structures of the significant loci.

## Materials and Methods

### Implementation

IntAssoPlot, built on ggplot2 (Wickham, 2016) R package, imports R packages including (Zheng et al., 2012), gdsfmt (Zheng et al., 2012, 2017), ggrepel^1^, and reshape2 (Wickham, 2007). Its basic functionality includes plotting GWAS results at the level of a single gene (IntGenicPlot) and a single chromosomal region (IntRegionalPlot). Owing to the characteristics of the R programming language (Ihaka and Gentleman, 1996), all these tools are open-source, and the package is easy to use and platform-free for bioinformaticians.

### Plot Methods

Modules provided by IntAssoPlot are listed and compared with other packages in Tables 1, 2, respectively. The output of IntAssoPlot contains three layers, integrated to display scatter plot of *P*-values for marker-trait associations, LD heat map of lead SNP (the most significant associated SNP or selected SNP) with the rest, LD heat map matrix, highlighted selected marker, and linking line (Table 1 and Supplementary Figure 1).

**TABLE 1:** Summary of functions implemented in IntAssoPlot.

Function	Layer	Shared modules	Differed modules
IntGenicPlot	Upper layer: a scatter plot displaying -log_10_(*P*) for marker-trait associations. Middle layer: Gene annotation. Bottom layer: LD matrix	Scatter plot of -log_10_(*P*) and marker site, LD heatmap of lead SNP (the most significant associated SNP or selected SNP) with the rest, LD matrix heatmap, highlight selected marker, linking line	Gene structure
IntRegionalPlot			Arrowed line reflecting gene length and strand

**TABLE 2:** Comparison of features provided in GWAS visualization tools.

Features in a single view	LocusZoom	cgmisc	Ldlink	Assocplots	IntAssoPlot
Scatter plot of marker-trait association	✓	✓	✓	✓	✓
Lead SNP LD heatmap	✓		✓		✓
LD matrix heatmap			✓		✓
Linking line from association to LD matrix					✓
LD matrix from another genotype dataset					✓
Gene structure	✓				✓
Integrated display of association, gene structure, and LD matrix					✓

Features of IntAssoPlot are compared with those of released software in Table 2. As shown in Table 2, IntAssoPlot could integrated display of marker-trait associations, gene structure, and LD matrix. Importantly, IntAssoPlot could facilitate genotype datasets different from those in GWAS to generate an LD matrix. Moreover, the color, shape, and size of approximately all of lead SNPs, highlighted markers, and LD heat map could be modified in IntAssoPlot, making the output more versatile and informative.

There are four inputs required for IntGenicPlot in IntAssoPlot, namely transcript, genome annotation file, GWAS result, and genotype file. For IntRegionalPlot, the chromosome, left border (start position), right border (end position), genome annotation file, GWAS result, and genotype file are required. The main panel of IntAssoPlot contains three layers and connecting lines. The upper layer shows *P*-values for association on the -log_10_ scale in the vertical axis. The middle layer presents the chromosomal position along the horizontal axis with the gene structure while the lower layer shows the LD matrix.

## Results and Discussion

### IntAssoPlot Workflow

R (The Comprehensive R Archive Network^2^) and RStudio (an integrated development environment for R^3^) should be downloaded and installed on the computer system. Currently, IntAssoPlot is hosted on GitHub. To download and build IntAssoPlot from GitHub, an R package devtools is required. Importantly, IntAssoPlot also imports some other R packages. When launching R, the depended packages, devtools, and IntAssoPlot can be installed using the following commands in R environment:

> install.packages(c(“devtools”,“ggplot2”,“ggrepel”,“reshape2”, “BiocManager”))
> BiocManager:install(c(“SNPRelate”,“gdsfmt”))
> devtools:install_github(“whweve/IntAssoPlot”,build = TRUE,build_opts = c(“–no-resave-data”, “–no-manual”))

To generate images by IntAssoPlot, there are two steps: (1) data input, (2) generate the image. Basically, IntAssoPlot needs three types of data, including association mapping results, gene annotation file, and genotype file, of which we have included detailed examples of these data in IntAssoPlot. When reading data into the R environment, R function read.table can be used. Below, we provide a detailed case study to illustrate the usage of IntAssoPlot.

### Case Study

**FIGURE 1:**
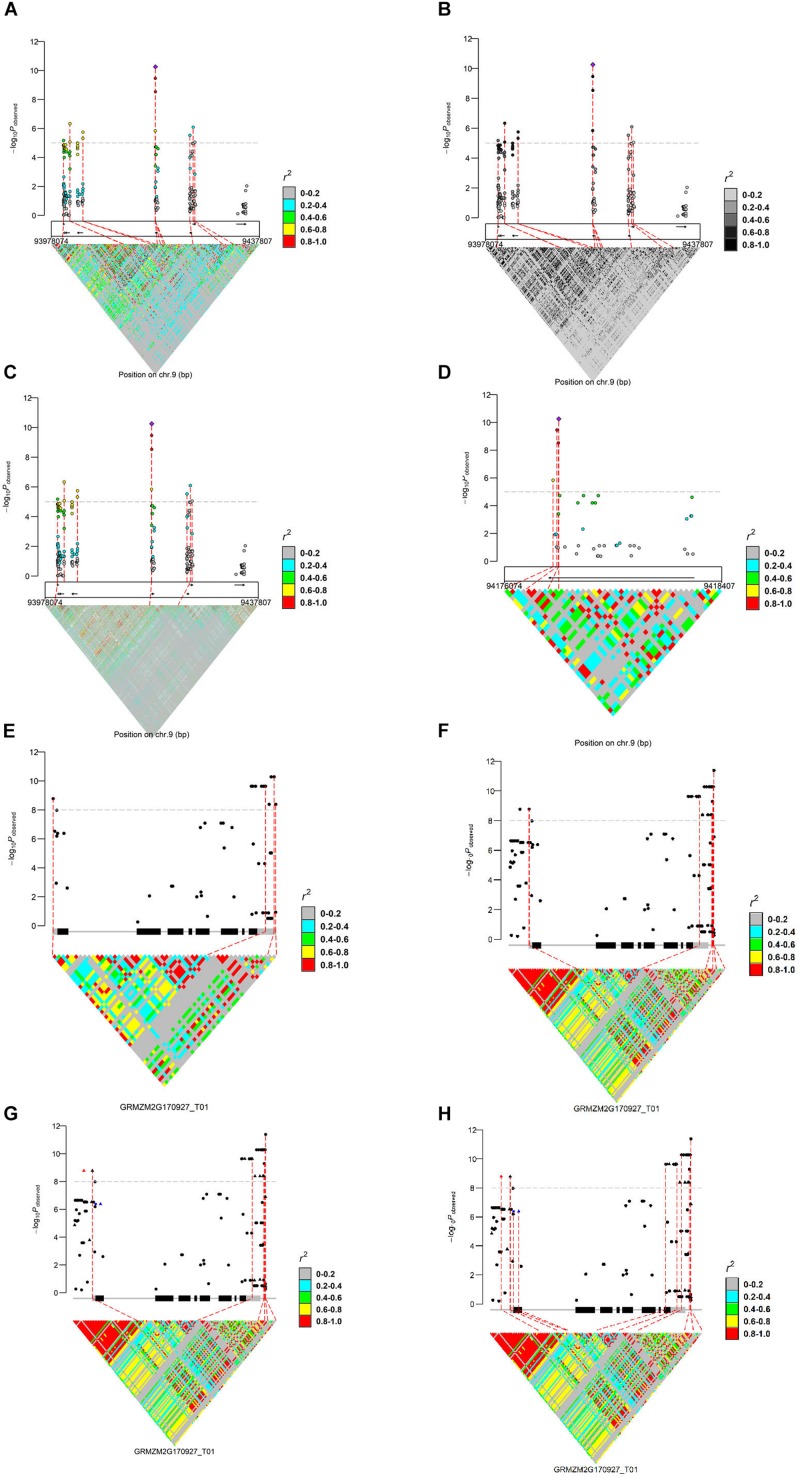
Regional marker-trait associations plot (**A:** regional plot; **B:** LD color scale; **C:** LD matrix from another genotypes; and **D:** zoom-out) and a single gene-based marker-trait associations plot (**E:** single genic plot; **F:** adjusting flanking sequence; **G:** highlighting markers; and **H:** linking markers), using previously published data (Wang et al., 2016). For **(A–D)**, the upper layer represents the marker-trait associations and LD of the most significant loci with the others; the middle layer shows the filtered gene models indicated by arrows (MaizeGDB release 5b.60); the bottom layer shows LD matrix. In **(E–H)**, the main transcript structure of *ZmVPP1* and the LD matrix are shown. Genotypes used to generate these plots were derived from Chia et al. (2012), Li et al. (2013), and Wang et al. (2016).

We present the features of IntAssoPlot and provide an example of the resulting plot (Figure 1) using previously published data on the genetic basis of maize drought tolerance in young maize seedlings (Wang et al., 2016), and we have included commands used to generate Figure 1 in Table 3.

**TABLE 3:** R commands used to generate Figure 1.

Scale	Operation	Command	Output
Regional plot	Lead SNP LD + triangle LD + directioned gene length + linking line	IntRegionalPlot(chr = 9,left = 94178074-200000,right = 94178074 + 200000,gtf = gtf,association = association,hapmap = hapmap_am368,hapmap_ld = hapmap_am368, threshold = 5, leadsnp_size = 2)	Figure 1A
	LD color scale	IntRegionalPlot(chr = 9,left = 94178074-200000,right = 94178074 + 200000,gtf = gtf, association = association,hapmap = hapmap_ am368,hapmap_ld = hapmap_am368, threshold = 5,leadsnp_size = 2,color02 = “gray81”,color04 = “gray61”,color06 = “gray41”,color08 = “gray11”,color10 = “gray1”)	Figure 1B
	Triangle LD from another set of genotype data	IntRegionalPlot(chr = 9,left = 94178074-200000,right = 94178074 + 200000,gtf = gtf, association = association,hapmap = hapmap _am368,hapmap_ld = hapmap2,threshold = 5, leadsnp_size = 2)	Figure 1C
	Zoom-in or -out	IntRegionalPlot(chr = 9,left = 94178074-2000,right = 94178074 + 6000,gtf = gtf, association = association,hapmap = hapmap_am368,hapmap_ld = hapmap_ am368,threshold = 5,leadsnp_size = 2)	Figure 1D
Single genic plot	Triangle LD + gene structure + linking line	IntGenicPlot(‘GRMZM2G170927_T01’, gtf,association = zmvpp1_association, hapmap = zmvpp1_hapmap,leadsnp = FALSE,triangleLD = TRUE,threshold = 8)	Figure 1E
	Flanking sequence	IntGenicPlot(‘GRMZM2G170927_T01’,gtf, association = zmvpp1_association,hapmap = zmvpp1_hapmap,leadsnp = FALSE, triangleLD = TRUE, threshold = 8,up = 500,down = 600)	Figure 1F
	Highlight marker by shape and/or color	IntGenicPlot(‘GRMZM2G170927_T01’,gtf, association = zmvpp1_association,hapmap = zmvpp1_hapmap,leadsnp = FALSE, triangleLD = TRUE,threshold = 8,up = 500, down = 600,marker2highlight = marker2highlight)	Figure 1G
	Selected markers linking from association to LD	IntGenicPlot(‘GRMZM2G170927_T01’,gtf, association = zmvpp1_association,hapmap = zmvpp1_hapmap,leadsnp = FALSE,triangleLD = TRUE,threshold = 8,up = 500,down = 600, marker2highlight = marker2highlight,link2gene = marker2link,link2LD = marker2link)	Figure 1H

Chromosomal regions can be selected in the following two ways: (1) the chromosome start and stop positions (Figures 1A–D) or (2) a transcript name (Figures 1E,F), with or without the specified marker. If no marker is specified, the most strongly associated marker will be treated as the lead SNP while a specified marker would be treated as the lead SNP, if provided. Available modules for displaying LD (*r*^2^ or *D*′) include lead SNP LD (leadsnpLD in IntAssoPlot) and triangle LD (triangleLD in IntAssoPlot). While the lead SNP LD function calculates LD of the most significant or user-provided loci with the others, the triangle LD function calculates the matrix of each pairwise LD from the specified region. All LD values are colored according to the specified color scale, which can be further modified, allowing the package to fit into broader applications (Figures 1A,B). Currently, researchers have released multiple sets of genotype data. In our package, genotype files for displaying lead SNP LD and association results were assumed to originate from the same set of genotype files. However, in order to take advantage of the massive genotype information, markers for displaying triangle LD could be derived from another set of genotype data (Figure 1C). The output image, generated by IntRegionalPlot, which is a function in IntAssoPlot, could be zoomed-in or -out (Figure 1D) by setting the length of left or/and right border. Meanwhile, IntGenicPlot allows us to show the association and LD related to flanking sequences, the length of which can be modified by the user (Figure 1F), thus revealing the effect of the promoter on the association results.

Notably, according to the input, linking lines and association signals can be selected and highlighted (Figures 1G,H), respectively. In IntGenicPlot and IntRegionalPlot, when setting a threshold for the genome-wide association significance level, a connecting line linking the significant association signals (removing completedly marker), gene structure, and LD matrix will be drawn by default (Figures 1A–G). If linking association signals are provided, linking lines can be reset accordingly (Figure 1H). Meanwhile, association signals in the upper layer could be highlighted by resetting the shape, color, and size (Figure 1G), thus making the output more useful.

Generally, generating an image by the function IntGenicPlot requires very limited time. However, for integrated visualization of GWAS results on a regional scale, the time is limited by the number of markers and the samples required to display the LD matrix. In the current example (Figure 1C), we have selected 2355 SNPs located over a region of 400 kbp in 104 samples (Chia et al., 2012) to display the LD matrix. Thus, 2,771,835 LD dots need to be displayed. When tested on a desktop computer with an Intel^®^ Core^TM^ i7-6700K processor and Windows 10 operation system, it was found to take less than 30 s to generate the integrated image, hence demonstrating the efficiency and performance of IntAssoPlot. However, <3 min will be consumed to print the image in the R environment. In Figures 1A,B, working with 368 samples and 270 SNPs, <30 s was required to generate and print the output image. Therefore, future studies should aim to reduce the time required to display the large LD matrix. It was reported that, based on undirected graphical models, netgwas can reconstructe LD networks adjusting for markers relationships (Behrouzi et al., 2017), which will be extended to IntAssoPlot in future development. On the other hand, by uploading IntAssoPlot at GitHub, we can make IntAssoPlot publicly accessible and invite the scientific community to contribute and enhance the capabilities of the package.

## Conclusion

At present, it is difficult to summarize GWAS results, genomic context, and linkage disequilibrium (LD) in a single view. The currently available tools cannot integrate, analyze, and display LD using genotypic markers different from those in association analysis. Therefore, we were prompted to present IntAssoPlot, an R package, aimed to solve these challenges. The output image of an IntAssoPlot plot, which is in a publication-ready format, draws a line connecting the *P*-values on a -log_10_ scale, the genome annotation, and the LD matrix, calculated from genotypes same or different from those in GWAS analysis. Inspection of the output image could reveal: (1) the extent of LD within a region with the most significant associated variants, providing important biological evidence to infer the candidate gene, (2) the most significantly associated genetic feature located within the candidate gene, and (3) the LD between the loci, demonstrating the mechanism of functional variation for further experimental validation.

Functions in IntAssoPlot are designed to be user-friendly, with attention to the quality of visualization, owing to the characteristics of R programming language. Documentation, outlining the detailed functionality, and parameter usage of each function, is available for each of the functions in IntAssoPlot. Detailed examples of the usage of IntAssoPlot have been created based on the analysis of data from a previously reported study on the genetic basis of maize drought tolerance (Wang et al., 2016). The IntAssoPlot package is available at https://github.com/whweve/IntAssoPlot.

## Data Availability Statement

The datasets generated for this study can be found in the https://github.com/whweve/IntAssoPlot.

## Author Contributions

FH, SD, and HW contributed to implementation, documentation, and manuscript writing. FQ contributed to conception, project design, oversight, and manuscript review. All authors read and approved the final manuscript.

## Conflict of Interest

The authors declare that the research was conducted in the absence of any commercial or financial relationships that could be construed as a potential conflict of interest.

## Footnotes

1 https://CRAN.R-project.org/package=ggrepel

2 https://cran.r-project.org/

3 https://www.rstudio.com/
