# Supplementary figures and images for: IntAssoPlot: An R Package for Integrated Visualization of Genome-Wide Association Study Results With Gene Structure and Linkage Disequilibrium Matrix
Source: Front Genet. 2020 Mar 20;11:260. doi: 10.3389/fgene.2020.00260 (PMC7100855; doi:10.3389/fgene.2020.00260)

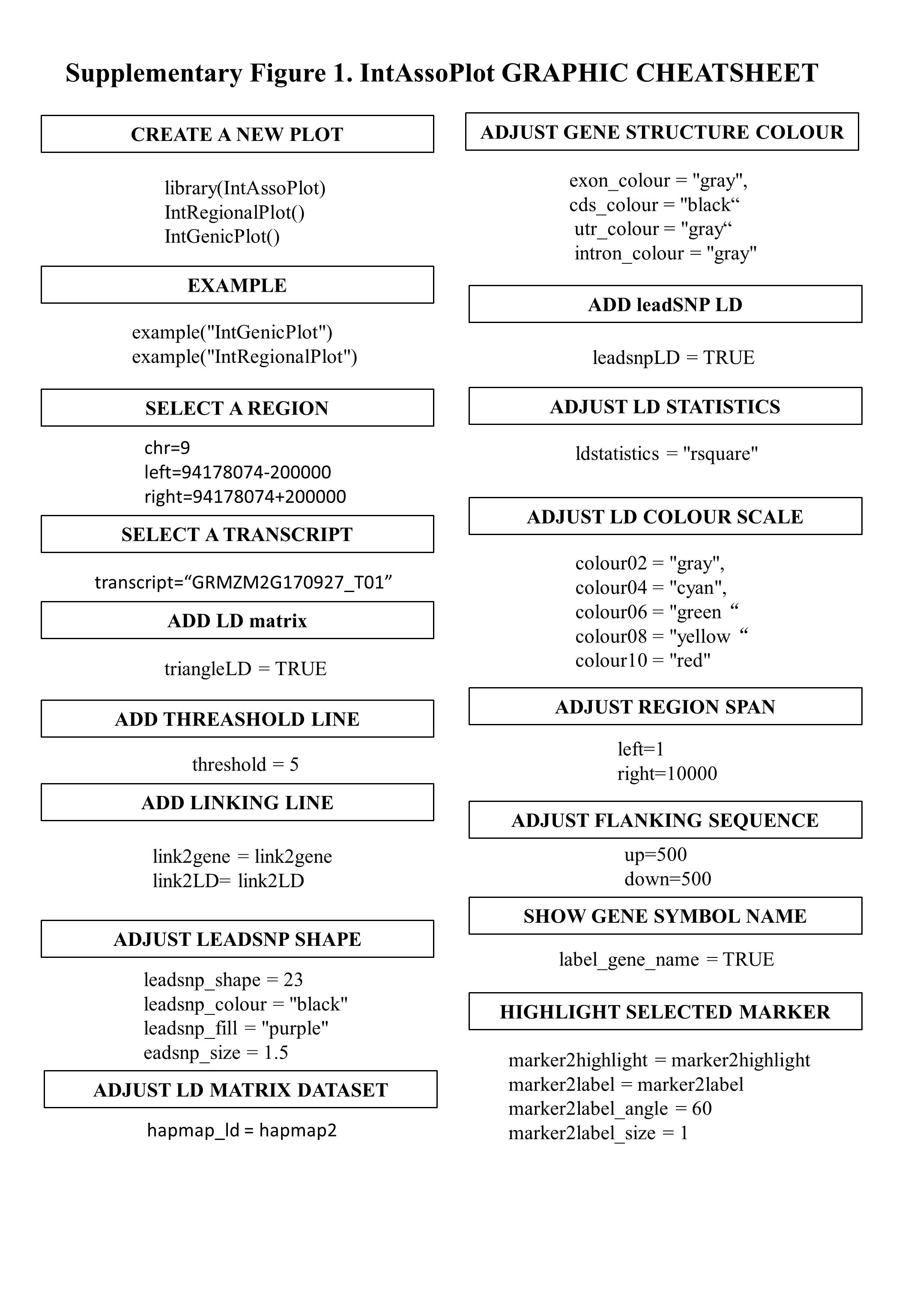

Supplement: Supplementary file 1 [file Image_1.jpg]
